# Measuring the synergy between technological and management innovation in megaprojects: Empirical evidence from China

**DOI:** 10.1371/journal.pone.0331330

**Published:** 2025-09-08

**Authors:** Yuanying Meng, Zhenxu Guo, Qing’e Wang, Lan Yin

**Affiliations:** 1 School of Highway, Chang’an University, Xi’an, People’s Republic of China; 2 Guangzhou Expressway Co., LTD, Guangzhou, People’s Republic of China; 3 School of Civil Engineering, Central South University, Changsha, People’s Republic of China; 4 Audit Bureau of Xi’an, Xi’an, People’s Republic of China; Yunnan University, CHINA

## Abstract

In the national innovation system, the synergistic development of technological and management innovation in megaprojects plays a critical role in achieving high-quality project delivery. This study aims to develop a quantifiable three-dimensional coordination measurement framework to explore the collaborative mechanisms between technological and management innovation and validate practical applicability. First, key influencing factors of both technological and management innovation are identified through a systematic literature review and expert interviews. Based on these factors, an innovation status measurement model is constructed using a combined subjective and objective weighting approach. Next, the coupling degree function is applied to assess the coordination levels of three subsystems: structural coordination, behavioral coordination, and performance matching. Moreover, the overall coordination degree is calculated by integrating these results using a power function. A case study of the Shuozhou-Huanghua Railway reveals that the structural coordination degree is low level, the behavioral coordination degree is medium level, the performance matching degree is medium level, and the overall coordination degree is medium level. These findings suggest that while collaboration at the behavioral level is relatively effective, there remains significant potential for improvement in structural and performance coordination. The proposed three-dimensional coordination framework and associated quantitative tools demonstrate strong diagnostic capability in evaluating the synergy, offering theoretical support for dynamic strategy formulation and resource optimization.

## 1. Introduction

In recent years, advanced technologies have been driving the engineering construction towards digital and green development [1–4]. This shift is especially critical for megaprojects, whose immense investment scales, technical complexity, and multitude of stakeholders render traditional methods insufficient [3,5–7]. For example, the Hong Kong-Zhuhai-Macao Bridge has achieved world-leading breakthroughs in deep-sea immersed tube tunnel technology [8,9] and enhanced system performance by integrating 5G technology and edge computing [10]. The “three-region collaboration” organizational management model [11] and BIM+ GIS digital management platform contribute to efficient cross-regional and multi-subject collaborative decision-making.

However, mismatches and conflicts persist due to the interplay between rapid technological advancements and existing management mechanisms, as well as between heterogeneous data integration challenges and organizational structure fragmentation [12, 13, 14, 15]. This leads to difficulties in quantifying and optimizing the synergy effect of the “technology-management” dual-wheel drive. To overcome this bottleneck, it is imperative to develop a systematic measurement framework that can quantitatively characterize the interaction gains between technological and management innovation and offer decision support for resource allocation and policy formulation, thereby unlocking the high-quality development potential of megaprojects.

The application of advanced technologies has not only transformed traditional engineering construction models but has also given rise to new management paradigms [16]. Previous studies have found that technological innovation must match management innovation [17], such as organization network [14], incentive and supervision mechanism [17, 18], and cooperation behavior [19]. In megaprojects, Chen and colleagues [20] and Rocha and colleagues [21] demonstrated that the deep integration of technology and management can enhance the quality and efficiency. Jin and colleagues [22] also found that the synergy of the two significantly enhanced sustainable development performance. However, most studies have focused on the assessment of a single dimension or merely described the positive correlation between the two through empirical cases, failing to provide operational tools for their collaborative measurement. Makarenko and Novosel’tsev [23] introduced a specific measure that serves to obtain some quantitative estimates of the synergy effect. Coombe and colleagues [24] defined and developed measures of partnership synergy as one dimension of a participatory mixed methods study. Garzella and Fiorentino [25] developed an effective synergy measurement model to support the decision-making process in mergers and acquisitions. Some scholars have also verified the synergy effect in areas such as mergers and acquisitions [26] and ecological conservation [27]. However, these frameworks are designed for enterprises or specific industries and fail to address the unique complexities of megaprojects, where there is a lack of multiple stakeholders, heterogeneous data sources, and fragmented management structures. Therefore, it is imperative to develop a systematic “technology-management synergy measurement framework” to quantify coupled efficiency, interaction, and performance. Based on these research gaps, this study aims to address the following questions:

Q1: What are the influencing factors of technological and management innovation for megaprojects?
Q2: How can the synergy model be constructed for subsystems and the whole system?
Q3: How can the quantified synergy between technological and management innovation be applied to support project management decisions, including strategic planning, resource allocation, and performance improvement?

The remainder of this study is organized as follows: Section 2: Literature Review is the literature review. Section 3: Methodology describes the research methodology and constructs the measurement model. Section 4: Case Study shows the measurement results of Shuozhou-Huanghua Railway. Section 5: Discussions discuss the results, and this study is summarized in Section 6: Conclusions.

## 2. Literature review

### 2.1. Collaborative innovation in megaprojects

Collaborative innovation is about the utilization of specific advantages of individual organizations in a holistic manner through collaboration for jointly solving management problems [28, 29]. Collaborative innovation contributes to sustainable development in many industries [30, 31]. Wang and Hu [32] reveal the mechanisms of collaborative innovation processes by investigating the complex relationships among critical factors influencing a firm’s innovation performance in supply chain networks. Companies increasingly seek to foster collaborative innovation through the design of innovation spaces [33]. Klooker and Holzle [34] agreed with this opinion.

Related research is also extended to construction projects [35]. Zhang and colleagues [36] explored complicated interaction relations of stakeholders in inter-organization collaborative innovation. Xue and colleagues [37] illustrated the importance of collaborative innovation relationships. In the context of collaborative innovation, technological and management innovation are important topics that must be discussed [38,39]. Damanpour [40] treat technological and management innovation equally in the dual-core innovation theory, arguing that both are equally important in enterprise innovation. Wang and colleagues [41] identified the influencing mechanism of technological and non-technological innovation. Zhao and colleagues [14] agreed that management innovation has enabled megaproject participants to engage significantly in technological innovation. These studies have gradually focused on the technological and management innovation of megaprojects. However, scholars rarely consider how technological and management innovation work together.

In recent years, digital transformation and AI technologies have begun to play a role in bridging the gap between technological and management innovation. By enabling real-time data processing, predictive analytics, and intelligent decision support, these technologies promote a closer integration of technical advancements with managerial processes [42, 43]. For example, AI-driven analytics can convert complex engineering data into actionable management insights, enabling project teams to align innovation outcomes with strategic objectives better [44,45]. Digital platforms further strengthen this synergy by integrating technological performance metrics with managerial control systems, ensuring that decisions regarding design, construction, and resource allocation are guided by both technical feasibility and managerial priorities [10,46]. It enhances the effectiveness of decision-making, thereby improving project performance in terms of long-term sustainability.

### 2.2. Collaborative system between technological and management innovation

The collaborative innovation of technological and management for megaprojects is a complex system, which is different from the enterprise [47], the industrial [48], and the regional innovation system [49]. It is worth exploring in depth to facilitate the successful delivery of megaprojects. In megaprojects, collaborative innovation denotes the process in which multiple stakeholders, such as owners, contractors, research institutions, and suppliers, utilize their resources, knowledge, and capabilities to advance both technological and management practices. Related studies on the mirror hypothesis theory indicate that a technological and management innovation collaborative system reflects a static structural relationship and a behavioral cooperative relationship [50]. “Structure” refers to the coupling degree between two innovation influencing factors. When technological innovation’s influencing factors change, management innovation’s elements will also change [51]. “Behavior” refers to the effectiveness between two innovation influencing factors. The coordinated development of technological and management innovation means the behavioral consistency [52]. Structure and behavior are analyzed from the perspective of process. From the perspective of results, matching performance needs to be deeply considered [53]. In conclusion, according to existing studies, collaborative innovation systems can be divided into three subsystems: structural coordination, behavioral cooperation, and performance matching. The three subsystems reflect the extent to which technological and management innovation drive and influence consistency in different dimensions. By integrating these three subsystems through a unified function into an overall synergy measure, it provides a clear, quantitative assessment of innovation interaction.

### 2.3. Synergy

Synergy, that is, the degree of cooperation, is employed to measure the degree of order of each subsystem, the degree of coordination and matching among subsystems, and the degree of coordination, seamless link, and efficient order of the system through self-organization evolution [54]. Synergy depends on the order degree of each subsystem and the degree of matching between subsystems. Haken [55] proposed that under certain conditions, through nonlinear interactions among subsystems, a synergetic effect can be generated, prompting the system to form a spatiotemporal self-organizing structure with specific functions [55]. Corning [56] further defined collaborative innovation as the overall effect of interacting multiple elements in a system. Based on this, scholars have carried out a series of studies on measuring synergy and put forward different evaluation methods. Koberg and colleagues [57] designed eight questions to measure the inter-departmental collaboration mechanism with the method of 5 points. Pinto and Guerreiro [58] assessed the synergy from four dimensions: economic structure, labor market, technological innovation, and human capital through factor analysis. Guo and Li [59]. measured the synergistic effect and innovation performance of the collaborative innovation of prefabricated construction enterprises based on the order parameter approach. The evaluation method of synergy has not been unified. To calculate the synergy of megaprojects, it is necessary to dig deeply to conform to the engineering practice.

## 3. Methodology

### 3.1. Scale design

Combined with the practice of megaprojects, this study quotes and adapts the typical problems of the technological innovation measurement items in the OECD Oslo Manual [60]). At the same time, adapting the conceptual framework of technological innovation influencing factors proposed by Camisón and Villar-López [61], this study determines four specific factors (i.e., TI1 technological innovation support elements, TI2 technological innovation planning, TI3 technological innovation communication and motivation, and TI4 technological innovation evaluation system). Regarding the management innovation items, this study referred to the research of [62–64]. The scale items were designed using the inductive method to ensure the accuracy of the item descriptions, considering the in-depth interviews with megaproject managers. Three influencing factors of management innovation (i.e., MI1 strategic coordination, MI2 organizational structure, and MI3 guarantee mechanism) were determined. A literature review selected a total of 30 items from the original scale that demonstrated excellent reliability (Cronbach’s α ≥ 0.80) and validity (standardized factor loadings ≥ 0.60). This study conducted Chinese-English translation and back-translation on these items and invited five experts with relevant research backgrounds to review the content validity. Four items with greater ambiguity were deleted. Through a pretest involving 30 project managers, the Cronbach’s α of all items was ≥ 0.75, and they were all significantly correlated with the original research items at above 0.70 (p < 0.001), which proved that the translated items were highly consistent with the original ones in meaning. Finally, 26 items were retained for the formal survey.

To ensure comprehensive sample coverage, this study distributed questionnaires to managers across 22 railway projects, 23 highway projects, 49 building projects, 33 bridge projects, and 17 port projects. The questionnaires adopted a 5-point Likert scale, where one indicated “very unimportant” and five indicated “very important.” A total of 175 valid questionnaires were collected, with an effective rate of 87.5%, meeting the requirement of confirmatory factor analysis that the number of observations should be at least 10 times the number of observed variables. The basic demographic characteristics of managers are presented in S1 Appendix.

**Fig 1:**
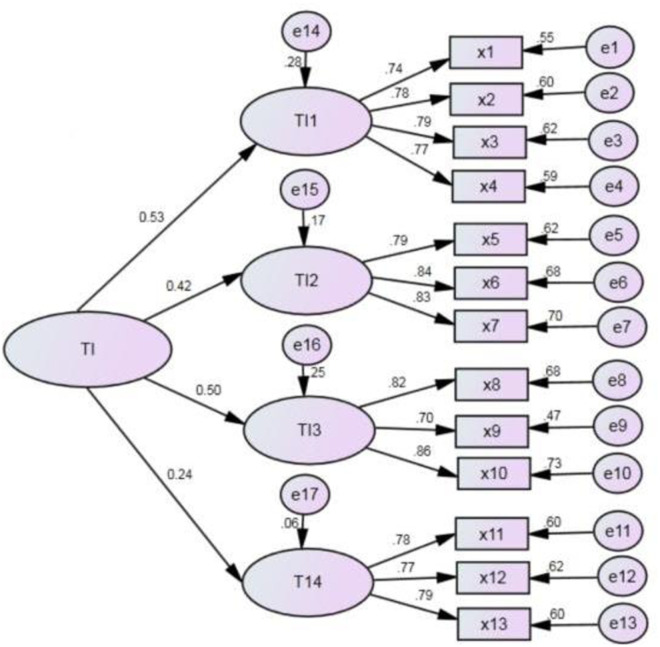
Confirmatory factor analysis model of technological innovation. In the model, the latent variable TI is composed of four first-order factors, TI1, TI2, TI3, and TI4, each of which is represented by several observed variables (x1 - x13). The path coefficients of all observed variables are above 0.7, indicating that each indicator has a strong explanatory power for its corresponding latent variable.

**Fig 2:**
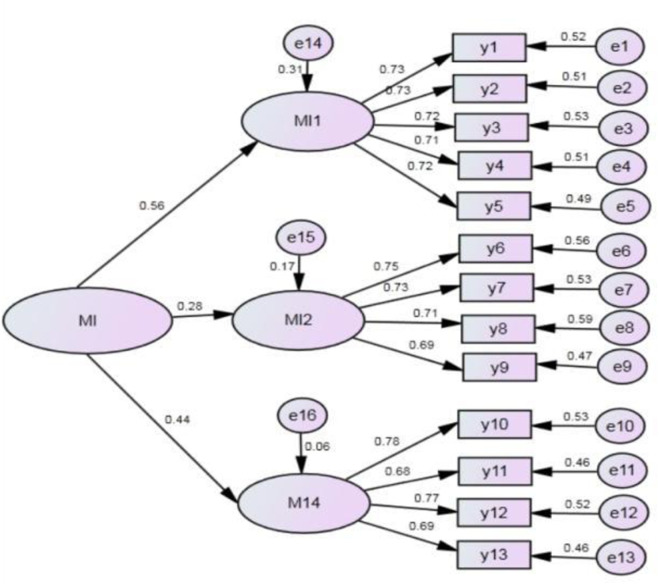
Confirmatory factor analysis model of management innovation. The latent variable MI is composed of three first-level factors, MI1, MI2, and MI4, each of which is measured by observed variables (y1-y13). The path coefficients are generally around 0.7, indicating that the observed variables have a good explanatory power for the latent variable.

Firstly, based on the SPSS software, reliability analysis was conducted on technological and management innovation scales. The internal consistency R^2^ coefficient of both was greater than 0.8, meeting the requirements. It indicates that the reliability and stability of the influencing factors are relatively high, allowing for confirmatory factor analysis. Secondly, standardized factor loading tests were carried out using the Amos software, obtaining the regression coefficients of the second-order confirmatory factor analysis model, as shown in Figs 1 and 2. All standardized factors of the observed variables under the four influencing factors of technological innovation exceeded 0.7 (exceeding 0.5 is considered satisfactory). Except for the standardized loading of the first-order influencing factor TI4 technological innovation evaluation system being significant at the 0.05 level, the standardized loading coefficients of other first-order influencing factors and their observed variables were significant at the 0.001 level. This indicates that the validity of the technological innovation influencing factors is good, and the model construction is consistent with the actual situation. Finally, the absolute fit index, incremental fit index, and parsimony fit index were calculated. The results show that the fit of the technological and management innovation model is good.

### 3.2. Construction of an innovation status measurement model

The term “state” originates from physics, referring to the condition of a material system. Megaproject innovation is constantly evolving, and innovation measurement can be defined as the actual description of the state in which megaproject innovation is situated. Previous studies have pointed out that measuring innovation status can be considered from the perspectives of innovation breadth and speed [65,66]. Innovation breadth is the cumulative number of achievements an enterprise obtains within a certain period [67]. It is difficult to illustrate all innovation achievements for megaproject innovation. The coverage of innovation activities is considered a more accurate and operational description of innovation breadth [65]. Innovation speed refers to the total time difference between the first adoption probability of innovation by the target and sample [66]. Considering the complexity and variability of megaproject innovation activities, this study consults experts in the field to determine the frequency of innovation within a given time as the value of innovation speed. The innovation breadth $I_{r}$ and innovation speed $I_{r}$ together constitute the measurement indicators of the innovation state $I=(I_{r},I_{s}{)}^{T}$. The innovation state value is comprehensively calculated by assigning weights to each measurement indicator.

This study employs a combined subjective and objective weighting approach to determine the weights of the influencing factors for technological and management innovation in megaprojects. Specifically, 10 experienced experts from house building, bridge, and railway projects evaluated the importance of each influencing factor index. A membership degree matrix was then constructed using the fuzzy evaluation method to calculate the subjective weights. Additionally, principal component and factor analysis were applied to reduce data dimension and extract key characteristic variables, thereby determining the objective weights. Based on the characteristics of megaprojects and expert opinions, the subjective weight coefficient is set to 0.6, and the objective weight coefficient is set to 0.4, a weighted combination of the subjective and objective weights is performed to derive the comprehensive weight values for each influencing factor [68]. The resulting comprehensive weights for technological innovation and management innovation factors are presented in Table 1.

**Table 1:** Comprehensive weight of influencing factors.

Indicator	Weight	Indicator	Weight	Indicator	Weight	Indicator	Weight
TI1	0.312	TI2	0.209	MI1	0.385	MI2	0.263
TI3	0.309	TI4	0.171	MI3	0.344	MI1	0.385
x1	0.206	x8	0.2634	y1	0.239	y8	0.249
x2	0.214	x9	0.3762	y2	0.188	y9	0.286
x3	0.293	x10	0.3604	y3	0.177	y10	0.232
x4	0.287	x11	0.335	y4	0.211	y11	0.219
x5	0.316	x12	0.320	y5	0.185	y12	0.269
x6	0.396	x13	0.345	y6	0.235	y13	0.280
x7	0.278	--	--	y7	0.230	--	--

Equations (1) and (2) show the formulas for measuring the breadth and speed of technological innovation. Based on the comprehensive weights. Equations (3) and (4) present the comprehensive measurement formulas for megaprojects $TI_{r}$ and $TI_{s}$.


$$TI_{r}=\sum_{i=1}^{4}\rho_{i}*TI_{ri}=\sum_{i=1}^{4}(\rho_{i}*\sum_{j=1}^{p}\delta_{ij}*\rho_{ij})$$ (1)

$$TI_{s}=\sum_{i=1}^{4}\rho_{i}*TI_{si}=\sum_{i=1}^{4}(\rho_{i}*\sum_{j=1}^{p}\eta_{ij}*\rho_{ij})$$ (2)

$$TI_{r}=\sum_{i=1}^{4}\rho_{i}*TI_{ri}=0.312TI_{r1}+0.209TI_{r2}+0.309TI_{r3}+0.171TI_{r4}$$ (3)

$$TI_{s}=\sum_{i=1}^{4}\rho_{i}*TI_{si}=0.312TI_{s1}+0.209TI_{s2}+0.309TI_{s3}+0.171TI_{s4}$$ (4)

**Note:** $\rho_{ij}$ represents the comprehensive weight value of the i-th indicator under the j-th influencing factor of technological innovation. p represents the total number of all indicators under the j-th influencing factor of megaprojects. $\eta_{ij}$ is the innovation speed score of the i-th indicator under the j-th influencing factor of technological innovation in mgaprojects.

The formulas for measuring management innovation’s breadth and speed are shown in formulas (5) and (6). According to the comprehensive weight, the comprehensive measurement formulas for megaprojects $MI_{r}$ and $MI_{s}$ are (7) and (8).


$$MI_{r}=\sum_{i=1}^{n}w_{i}*MI_{ri}=\sum_{i=1}^{n}(w_{i}*\sum_{j=1}^{m}w_{ij}*r_{ij})$$ (5)

$$MI_{s}=\sum_{i=1}^{n}w_{i}*MI_{si}=\sum_{i=1}^{n}(w_{i}*\sum_{j=1}^{m}w_{ij}*s_{ij})$$ (6)

$$MI_{r}=\sum_{i=1}^{3}w_{i}*MI_{ri}=0.385MI_{r1}+0.263MI_{r2}+0.344MI_{r3}$$ (7)

$$MIs=\sum_{i=1}^{3}w_{i}*MI_{si}=0.385MI_{s1}+0.263MI_{s2}+0.344MI_{s3}$$ (8)

**Note:** $s_{ij}$ represents the comprehensive weight value of the i-th indicator under the j-th influencing factor of management innovation. m represents the total number of all indicators under the j-th influencing factor of megaprojects. $w_{ij}$ is the innovation speed score of the i-th indicator under the j-th influencing factor of management innovation in mgaprojects.

### 3.3. Construction of synergy measurement model

#### 3.3.1. The synergy model for the structural coordination subsystem.

Structural coordination in technological and management innovation lies in the duality among influencing factors. Based on relevant theoretical research and the comprehensive literature review, this study employs the coupling degree function to measure the structural coordination subsystem of technological and management innovation. The coupling degree model concept integrates four influencing factors of technological innovation and the three influencing factors of management innovation into the coupling measurement model. Based on the initial formula (9), the measurement formula (10) for the synergy of the structural coordination subsystem is derived.


$$\left\{ \begin{array}{c} C_{TI_{i}-MI_{j}}=\frac{2\sqrt{TI_{i}*MI_{j}}}{TI_{i}+MI_{j}}\\ F_{s}={\left[\prod C_{TI_{i}-MI_{j}}\right]}^{7}\\ TI_{i}=T{I_{i}}_{r}*T{I_{i}}_{s}\\ MI_{j}=M{I_{j}}_{r}*M{I_{j}}_{s}\\ i=1,2,3,4\\ j=1,2,3 \end{array}\right.$$ (9)

$$F_{s}=\prod {\left[\frac{2\sqrt{TI_{i}*MI_{j}}}{TI_{i}+MI_{j}}\right]}^{7}$$ (10)

**Fig 3:**
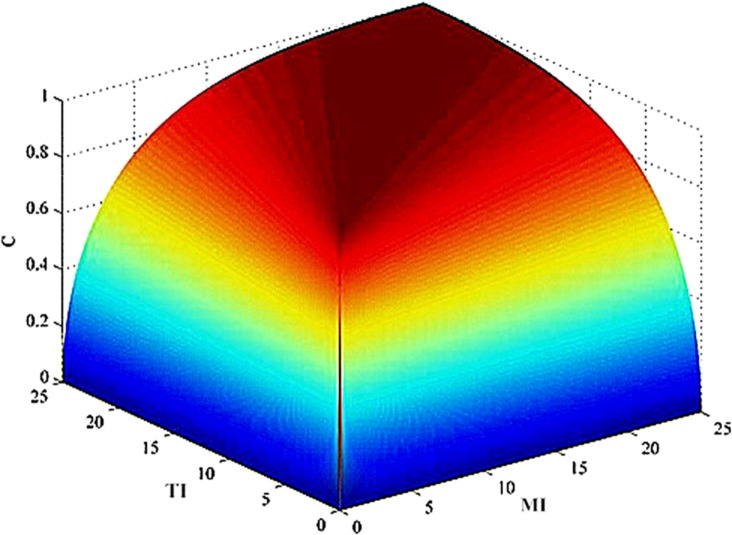
Simulation results of the synergy for the structural coordination subsystem. TI represents the influencing factors of technological innovation, MI represents the influencing factors of management innovation, and C represents the coordination degree between the two. The overall surface is curved, with its highest point close to C = 1 and the lowest point close to C = 0. In terms of color representation, the surface gradually transitions from bottom to top, changing from blue (low coordination) to red (high coordination), intuitively reflecting the differences in the degree of synergy between TI and MI at different levels.

Based on the data, a Matlab simulation was performed, and the resulting range of $C_{TI_{i}-MI_{j}}$ is presented in Fig 3. By applying $C_{TI_{i}-MI_{j}}$ successive multiplication, it is determined that the value range lies within (0, 1]. Consequently, the function $F_{s}$ exhibits monotonic decreasing behavior, $0<F_{s}\leq 1$. The simulation results indicate that the coordination degree of the structural coordination subsystem can be categorized into three levels: low ($0<F_{s}<0.4$), medium ($0.4<F_{s}\leq 0.6$), and high ($0.6<F_{s}\leq 1$).

#### 3.3.2. The synergy model for the behavioral cooperation subsystem.

There is a high degree of consistency between technological and management innovation at the behavioral level. The behavioral cooperation between them can be quantified by the difference vector between them. This difference vector consists of the differences in innovation breadth and innovation speed and is represented as a vector pointing from management innovation toward technological innovation. Therefore, the difference vector for the behavioral cooperation subsystems is $MT=(MI_{r},MI_{s}{)}^{T}-(TI_{r},TI_{s}{)}^{T}=(MT_{r},MT_{s}{)}^{T}$, the absolute value is $v=\sqrt{{\left(MI_{r}-TI_{r}\right)}^{2}+{\left(MI_{s}-TI_{s}\right)}^{2}}$. The larger the absolute value of the difference vector, the lower the synergy of TI and MI. Suppose the synergy of these subsystems is denoted by $F_{b}$. In that case, it indicates that $F_{b}$ and v are negatively correlated functions, as shown in formula (11).


$$\left\{ \begin{array}{c} F_{b}=e^{-v}=e^{-{\left[{\left(MI_{r}-TI_{r}\right)}^{2}+{\left(MI_{s}-TI_{s}\right)}^{2}\right]}^{\frac{1}{2}}}\\ TI_{r}=\sum_{i=1}^{4}\rho_{i}*TI_{ri}\\ TI_{s}=\sum_{i=1}^{4}\rho_{i}*TI_{si}\\ MI_{r}=\sum_{i=1}^{3}w_{i}*MI_{ri}\\ MI_{s}=\sum_{i=1}^{3}w_{i}*MI_{si} \end{array}\right.$$ (11)

In the above formula, the range of values for the exponential function with base e is (0, 1); therefore, the range of values for $F_{b}$ is also (0, 1). Based on the questionnaire data, the synergy of the behavioral cooperation subsystem is categorized into three levels: low level ($0<F_{b}<0.3$), medium level ($0.3\leq F_{b}<0.7$), and high level ($0.7\leq F_{b}<1$).

#### 3.3.3. The synergy model for the performance matching subsystem.

According to the existing literature, the performance matching between technological and management innovation can be examined from two key dimensions: effectiveness and sustainability. Based on the practice of megaprojects, three items were developed from the perspective of innovative achievements to evaluate effectiveness [10,69]. The sustainability indicators reflect the standardization of innovative achievements, the identification of input and output of innovation achievements, and the improvement of innovation capabilities in megaprojects [70,71]. Through the questionnaire survey, three items were designed to represent the sustainability dimension of technological and management innovation. Experts scored the performance matching indicators, enabling the construction of a judgment matrix based on these scores. Subsequently, the eigenvector and eigenvalue were calculated. The consistency index (C.I.) and the random consistency ratio (C.R.) satisfied the requirements, leading to the final determination of the weights for the performance matching, as presented in Table 2.

**Table 2:** Performance matching indicators and weights.

Indicator	Items	Weight
Effectiveness	E1 Megaprojects innovation achievements have been effectively integrated into engineering practice.	0.23
	E2 The quantity of intellectual property rights generated by megaprojects.	0.13
	E3 The growth status of economic benefits in megaprojects.	0.14
Sustainability	E4 The standardization level of innovation achievements in megaprojects.	0.12
	E5 The identification of input and output of innovation achievements in megaprojects.	0.22
	E6 The improvement of innovation capabilities in megaprojects.	0.16

Based on the weight calculation results presented in Table 2, the measured value of the synergy for the performance matching subsystem of technological and management innovation $F_{e}$ was derived, as indicated in Formula (12).


$$F_{e}=\frac{1}{6}(0.23E_{1}+0.13E_{2}+0.14E_{3}+0.12E_{4}+0.22E_{5}+0.16E_{6})$$ (12)

This study collects data using a five-point Likert scale in questionnaire surveys. The synergy measurement value of the performance matching subsystem for technological and management innovation falls within the range (0, 1). Based on the questionnaire data, the synergy is categorized into three levels: low level ($0<F_{e}<0.55$), medium level ($0.55\leq F_{e}<0.7$), and high level ($0.7\leq F_{e}<1$).

#### 3.3.4. The overall synergy model.

The synergy measurement of the structural coordination, behavioral cooperation, and performance matching subsystems represent the collaborative characteristics of the technological and management innovation system at different levels, satisfying the multi-level and non-linear nature. In this paper, the product of these three subsystems is processed using a power exponential function to construct the integrated measurement model for the synergy. The overall synergy measurement is presented in Formula (13).


$$\left\{ \begin{array}{c} F_{TI-MI}=(Fs*Fb*Fe{)}^{\frac{1}{3}}\\ F_{s}={\left[\prod \left[\frac{2\sqrt{MI_{i}*TI_{j}}}{MI_{i}+TI_{j}}\right]\right]}^{\frac{1}{7}}\\ F_{b}=e^{-v}=e^{-{\left[{\left(MI_{r}-TI_{r}\right)}^{2}+{\left(MI_{s}-TI_{s}\right)}^{2}\right]}^{\frac{1}{2}}}\\ F_{e}=\frac{1}{6}\sum_{i=1}^{6}\lambda_{i}*E_{i} \end{array}\right.$$ (13)

To measure the overall synergy, the EXP function operation is utilized, and cluster analysis is performed using SPSS software. By integrating the cluster center values with the value range of the sample classification criteria, the comprehensive measurement value for the synergy between technological and management innovation $F_{TI-MI}$ is derived. The overall synergy degree can be categorized into three levels: low level ($0<F_{TI-MI}\leq 0.5$), medium level (0.5<*F*_*TI-MI*≤0.65_), and high level ($0.65\leq F_{TI-MI}<1$).

## 4. Case study

### 4.1. Case context

The Shuozhou-Huanghua Railway extends westward from Shenzhi South Station in Shanxi Province and eastward to Huanghua Port Station in Hebei Province. The mainline spans approximately 594 kilometers, with 33 stations along the route. As a critical west-to-east coal transportation corridor in China, the Shuozhou-Huanghua Railway accounts for 10% of the nation’s total coal transportation volume and 20% of the west-to-east coal transportation share. It serves as a key railway artery ensuring the country’s energy supply. Jointly constructed by Shenhua Group, the former Ministry of Railways, and Hebei Province, the Shuozhou-Huanghua Railway Company is responsible for construction organization and operational management. Since full electrification and opening to traffic in November 2002, the Shuozhou-Huanghua Railway has achieved significant technological and management innovation accomplishments, establishing itself as a benchmark for the high-quality development of megaprojects.

The technological innovation of the Shuozhou-Huanghua Railway has been realized through the integration of internal and external resources, including technology, talent, equipment, and funding from Shenhua Group. By leveraging the innovation platform and collaborating with partner organizations such as the China Academy of Railway Sciences, it has successfully addressed key technical challenges, such as 30-ton axle load transportation. Additionally, it has expanded its funding sources via the National Science and Technology Support Program, increasing R&D investment from 0.8% in 2010 to 2.3%. The innovation strategy is guided by engineering requirements, focusing on capacity expansion and renovation projects for railways like the Shuozhou-Huanghua Railway, aiming to achieve an annual transportation volume exceeding 400 million tons. Shenhua Group leads the allocation of benefits and risk-sharing mechanisms. It promotes innovation through salary-linked incentives and diverse rewards, such as the “Five Minor” innovation awards (minor inventions, minor innovations, minor renovations, minor designs, minor suggestions). Furthermore, it has established a comprehensive group-level technological innovation evaluation system based on economic value-added principles. This system emphasizes practical services, reasonable indicators, scientific procedures, long-term considerations, and capability enhancement. It quantitatively assesses performance across dimensions such as investment, benefits, and intellectual property rights to ensure the achievement of technological objectives.

National strategic demands and corporate objectives jointly drive the management innovation of the Shuozhou-Huanghua Railway. It is promoted through “systematic planning, project-based support, synchronized implementation, and international cooperation.” At the strategic level, the railway focuses on “high axle load, high traction mass, and high operational density,” concentrating on the research and development of integrated technology systems, locomotive and vehicle control technologies, and advanced communication technologies. It adopts a cooperative innovation model and forms strategic alliances to enhance management innovation capabilities. Regarding organizational structure, the Shuozhou-Huanghua Railway has established an innovation network centered around leading users, integrating resources from research institutions, universities, and suppliers, forming a multi-level management system to strengthen internal and external collaboration. Regarding guarantee mechanisms, the railway optimizes the entire process from project initiation and implementation to acceptance by improving the scientific research management system, highlighting intellectual property management, enhancing innovation process control, and leveraging contractual agreements to strengthen supervision and post-evaluation, ensuring the practical realization of technological innovation goals.

### 4.2. Innovation status measurement

This study conducted interviews and surveys with five managers involved in the construction and operation of the Shuozhou-Huanghua Railway for over a decade. Based on the interview findings and questionnaire responses, along with the formulas presented in Section 3.3 and the weight data provided in Table 1, the technological and management innovation status of the Shuozhou-Huanghua Railway was calculated, as illustrated in Table 3.

**Table 3:** Innovation status measurement results.

Indicator	$T{I_{ri}}^{s}$	$T{I_{si}}^{s}$	Indicator	$MI_{ri}^{s}$	$MI_{si}^{s}$
TI1	4.226	3.631	MI1	3.426	3.364
TI2	3.676	3.928	MI2	3.317	3.228
TI3	3.876	3.414	MI3	3.326	3.414
TI4	3.521	4.364	MI	3.301	3.212
TI	3.511	3.382	–	–	–

Table 3 indicates that the breadth values of technological innovation, ranked from highest to lowest, are support elements for technological innovation, communication and motivation in technological innovation, planning for technological innovation, and the evaluation system for technological innovation. The speed values of technological innovation, ranked from highest to lowest, are the evaluation system for technological innovation, planning for technological innovation, support elements for technological innovation, and communication and motivation in technological innovation. For management innovation, the breadth values, ranked from highest to lowest, are strategic coordination, guarantee mechanisms, and organizational structure. The speed values of management innovation, ranked from highest to lowest, are guarantee mechanisms, strategic coordination, and organizational structure.

### 4.3. Synergy measurement

This study sequentially calculates the coordination degrees of the structural coordination, behavioral cooperation, and performance matching subsystem for both technological and management innovation of the Shuozhou-Huanghua Railway and the overall synergy. By combining formula (10) with the results of the innovation state measurement, the value of the structural coordination subsystem for technological and management innovation at Shuozhou-Huanghua Railway $F_{s}^{s}$ is calculated to be 0.327. According to the classification of synergy levels for the structural coordination subsystem, Shuohuang Railway falls within the low-level structural coordination range. This indicates that the relationships among the factors that influence technological and management innovation at Shuozhou-Huanghua Railway are relatively loose and poorly coordinated. Based on the innovation state values presented in Section 4.2, the difference between technological and management innovation is calculated to be −0.367. From a vector direction perspective, technological innovation is dominant, which aligns with the vigorous implementation of the technological innovation strategy in Shuohuang Railway’s practice. Using the formula (11), the value of the behavioral cooperation subsystem $F_{b}^{s}$ is calculated to be 0.6928, suggesting that the behavioral consistency of technological and management innovation at Shuozhou-Huanghua Railway is at a medium level. According to formula (12), the performance matching subsystem value $F_{e}^{s}$ is calculated to be 0.6522, indicating a medium level of efficiency alignment. By substituting $F_{s}^{s}$, $F_{b}^{s}$, and $F_{e}^{s}$ into formula (13), the overall synergy is obtained as 0.5294, reflecting the medium level.

## 5. Discussions

### 5.1. Collaborative-driven strategy formulation

The coordinated development of technological and management innovation is a critical driving force for implementing megaproject innovation. This perspective contrasts with the findings of [13], who argues that companies fail to achieve better outcomes by introducing technological and management innovations concurrently. Technological Communication and Motivation (TI3) enhances Organizational Structure (MI2). This improved organizational alignment subsequently reinforces the Technological Innovation Evaluation System (TI4), forming a positive feedback loop that accelerates both the breadth and speed of innovation. Meanwhile, robust Guarantee Mechanisms (MI3) improve the accuracy and responsiveness of the evaluation system (TI4), ensuring that performance outcomes effectively drive subsequent planning (TI2) and resource allocation (TI1). Although our current framework does not explicitly quantify these causal relationships, the qualitative path analysis identifies critical feedback mechanisms that support the observed levels of coupling and coordination.

Strategic, coordinated planning enables the systematic advancement of technological and management innovation activities, significantly improving the management efficiency and technological innovation efficiency of megaprojects while enhancing the competitiveness of engineering innovation. In strategic formulation, the following aspects warrant attention: First, a medium-term and short-term coordinated development goal system should be established to clarify the implementation pathways and operational procedures for technological and management innovation. Second, from a systems engineering perspective, the correlation mechanism between technological and management innovation should be thoroughly analyzed, and the resource requirements and potential barriers during implementation should be systematically assessed. Third, key factors influencing the synergy between them should be comprehensively evaluated, and the integrated advantages of megaprojects, such as technological accumulation, management systems, talent reserves, and financial support, should be fully leveraged in conjunction with scientifically informed predictions of environmental dynamics and complexity.

### 5.2. Innovation status diagnosis and analysis

Measurement data indicates that technological innovation’s breadth and speed significantly surpass management innovation. This disparity is primarily manifested in the higher maturity levels of the four critical elements of technological innovation (supporting factors, communication and incentive mechanisms, planning systems, and evaluation frameworks), which outperform their corresponding dimensions in management innovation. Consequently, the structural coordination subsystem currently operates at a low level, whereas the behavioral cooperation subsystem achieves a medium level. To enhance the synergy between technological innovation and management innovation in megaprojects, it is essential to systematically diagnose the current state of both types of innovation, construct an innovation element correlation map for quantitatively analyzing the interaction intensity among influencing factors, and combine this with engineering practice to develop an evaluation system for the implementation effects of “dual innovation.” Additionally, quarterly dynamic monitoring should be implemented based on changes in the innovation environment, and comparative analysis should be conducted to identify deviations between the current state and strategic objectives. This process will provide a scientific basis for decision-making regarding innovation resource allocation and enhancing synergy in subsequent stages. The diagnostic framework offers a benchmark for adjusting innovation strategies in megaprojects and extends methodological applicability to similar innovation management practices.

### 5.3. Dynamic control of collaborative innovation

The technological and management innovations of megaprojects exhibit significant dynamic coupling characteristics as the breadth and speed of innovation continuously evolve in response to project progress and environmental changes. Megaprojects must comprehensively account for the instability of engineering demands, adjustments in organizational structure, iterative updates of construction techniques, abrupt shifts in technical routes, and fluctuations in the supply chain system [72]. This study proposes a three-stage dynamic control model based on the research findings: “monitoring - comparison - regulation.” In the first stage, a dual-dimensional innovation index monitoring system is established to quantify the completion progress of indicators such as technology research and development and process improvement and to evaluate the implementation effects of dimensions like organizational structure and process reengineering. A coordination procedure is initiated if the deviation in innovation progress for either the technological or management dimension exceeds the threshold. In the second stage, a dual-comparison analysis method is employed to compare the actual innovation trajectory and the predefined collaborative development strategy. Technological innovation is aligned with management process reengineering, while technological enhancement is driven by management requirements to achieve horizontal coupling. In the third stage, dynamic optimization of innovation resource allocation is implemented based on assessing environmental uncertainty. A feedback loop for evaluating the degree of collaboration is established, and the innovation synergy effect is regularly assessed to adjust the implementation strategy dynamically.

## 6. Conclusions

This study focuses on megaprojects as the research object. It constructs an innovation status measurement model by systematically analyzing the interaction mechanism between technological and management innovation. A three-dimensional analysis framework for the technological and management innovation system is established, comprising three subsystems: structural coordination, behavioral cooperation, and performance matching. Specifically, the structural coordination subsystem evaluates the degree of association among influencing factors of technological and management innovation; the behavioral cooperation subsystem analyzes the differences in implementation breadth and speed between the two types of innovation. The performance matching subsystem measures the contribution of both innovations to achieving project goals. This study proposes a systematic method for measuring synergy. A synergy model for the structural coordination subsystem is constructed based on the measurement of influencing factor weights. According to the results of innovation status measurements, an analysis model for the behavioral cooperation subsystem is developed. From the perspective of system output, an evaluation model for the performance matching subsystem is designed. On this basis, the measurement results of the three subsystems are integrated to construct a calculation model for the overall system synergy. Finally, an empirical study of the Shuozhou-Huanghua Railway verifies the effectiveness and applicability of the proposed model. The empirical results demonstrate that the measurement model can effectively assess the synergy status of technological and management innovation in megaprojects.

This study investigates measuring the homogenization state of different types of innovation in megaprojects. Its main contributions are as follows: First, it develops a systematic framework for quantitatively measuring innovation, addressing the gaps in existing research. Second, it illustrates the complex interrelationship mechanism between technological and management innovation. Third, these findings provide a scientific foundation for evaluating the innovation level of megaprojects, offer guidance for formulating innovation management decisions, and facilitate the achievement of innovation goals in megaprojects.

This study acknowledges the following limitations and suggests corresponding future research directions. First, the measurement model is primarily validated on large infrastructure projects including railways, roads, buildings, bridges, and ports. Its generalizability to distinct sectors such as IT or pharmaceutical development, or smaller-scale projects like community renovations, requires rigorous verification. This study aims to broaden the scope of the questionnaire and interview participants from exclusively project managers to include project stakeholders across different hierarchical levels and functional departments, thereby obtaining more comprehensive and objective data. Second, the static analysis fails to capture dynamic variations in innovation synergy across critical project life cycle phases, particularly between initial planning and active construction stages. Future longitudinal studies should examine how evolving contextual factors temporally influence synergistic effects. Third, while identifying key drivers, the causal mechanisms underlying technological-management innovation synergy remain unexamined. Future research must empirically test interaction pathways through methods such as structural equation modeling and system dynamics. Finally, establishing explicit links between achieved innovation synergy and key performance metrics, including cost efficiency, schedule adherence, safety compliance, and sustainability outcomes, is essential for advancing both theoretical frameworks and industry practices.

## Supporting information

S1 Appendix Demographic Characteristics. (DOCX)

S1 Data Minimal Data Set. (DOCX)
