# Supplementary material for: Measuring the synergy between technological and management innovation in megaprojects: Empirical evidence from China
Source: PLoS One. 2025 Sep 8;20(9):e0331330. doi: 10.1371/journal.pone.0331330 (PMC12416728; doi:10.1371/journal.pone.0331330)
Supplement: S1 Appendix — (DOCX) [file pone.0331330.s001.docx]

**Appendix 1: Demographic Characteristics**

| Variable | Category | Number | Percentage(100%) |
| --- | --- | --- | --- |
| Age (years) | 25-34 | 58 | 33.1 |
|  | 35-44 | 80 | 45.7 |
|  | ≥45 | 37 | 21.2 |
| Education Level | Bachelor’s degree below | 40 | 22.9 |
|  | Bachelor’s degree | 95 | 54.3 |
|  | Master’s degree or above | 40 | 22.9 |
| Years of Experience | <5 | 30 | 17.1 |
|  | 5-10 | 70 | 40.0 |
|  | >10 | 75 | 42.9 |
