# Supplementary material for: Measuring the synergy between technological and management innovation in megaprojects: Empirical evidence from China
Source: PLoS One. 2025 Sep 8;20(9):e0331330. doi: 10.1371/journal.pone.0331330 (PMC12416728; doi:10.1371/journal.pone.0331330)
Supplement: S1 Data — (DOCX) [file pone.0331330.s002.docx]

**Minimal Data Set**

Table1. Parameter estimation of the model of influencing factors of technological innovation.

| Model regression path | | | Unstandardized parameter estimates | S.E. | C.R. | Sig. | Standardized parameter estimates |
| --- | --- | --- | --- | --- | --- | --- | --- |
| TI_1_ | <--- | TI | 0.338 | 0.083 | 3.725 | *** | 0.527 |
| TI_2_ | <--- | TI | 0.245 | 0.067 | 3.363 | *** | 0.421 |
| TI_3_ | <--- | TI | 0.307 | 0.089 | 3.657 | *** | 0.496 |
| TI_4_ | <--- | TI | 0.116 | 0.064 | 2.124 | 0.043 | 0.240 |
| X_1_ | <--- | TI_1_ | 1.000 | —— | —— | —— | 0.742 |
| X_2_ | <--- | TI_1_ | 0.925 | 0.086 | 10.276 | *** | 0.778 |
| X_3_ | <--- | TI_1_ | 1.032 | 0.103 | 10.443 | *** | 0.791 |
| X_4_ | <--- | TI_1_ | 0.853 | 0.089 | 10.214 | *** | 0.768 |
| X_5_ | <--- | TI_2_ | 1.000 | —— | —— | —— | 0.789 |
| X_6_ | <--- | TI_2_ | 1.043 | 0.090 | 11.685 | *** | 0.842 |
| X_7_ | <--- | TI_2_ | 1.032 | 0.093 | 11.802 | *** | 0.827 |
| X_8_ | <--- | TI_3_ | 1.000 | —— | —— | —— | 0.823 |
| X_9_ | <--- | TI_3_ | 0.842 | 0.088 | 9.802 | *** | 0.692 |
| X_10_ | <--- | TI_3_ | 0.945 | 0.084 | 11.008 | *** | 0.861 |
| X_11_ | <--- | TI_4_ | 1.000 | —— | —— | —— | 0.776 |
| X_12_ | <--- | TI_4_ | 0.965 | 0.095 | 9.798 | *** | 0.773 |
| X_13_ | <--- | TI_4_ | 0.976 | 0.099 | 9.861 | *** | 0.792 |

Table 2. Subjective weights of influencing factors of technological innovation.

| Indicator | Weight | Indicator | Weight | Indicator | Weight | Indicator | Weight |
| --- | --- | --- | --- | --- | --- | --- | --- |
| TI_1_ | 0.314 | TI_2_ | 0.185 | TI_3_ | 0.311 | TI_4_ | 0.190 |
| X1 | 0.179 | X5 | 0.317 | X8 | 0.215 | X11 | 0.336 |
| X2 | 0.186 | X6 | 0.422 | X9 | 0.421 | X12 | 0.312 |
| X3 | 0.318 | X7 | 0.261 | X10 | 0.364 | X13 | 0.352 |
| X4 | 0.317 | -- | -- | -- | -- | -- | -- |

Table 3. Objective weights of influencing factors of technological innovation.

| Indicator | Weight | Indicator | Weight | Indicator | Weight | Indicator | Weight |
| --- | --- | --- | --- | --- | --- | --- | --- |
| TI1 | 0.308 | TI2 | 0.246 | TI3 | 0.305 | TI4 | 0.142 |
| X1 | 0.247 | X5 | 0.315 | X8 | 0.336 | X11 | 0.333 |
| X2 | 0.256 | X6 | 0.342 | X9 | 0.309 | X12 | 0.331 |
| X3 | 0.255 | X7 | 0.303 | X10 | 0.355 | X13 | 0.336 |
| X4 | 0.242 | -- | -- | -- | -- | -- | -- |

Table 4. Parameter estimation of the model of influencing factors of management innovation.

| Model regression path | | | Unstandardized parameter estimates | S.E. | C.R. | Sig. | Standardized parameter estimates |
| --- | --- | --- | --- | --- | --- | --- | --- |
| MI1 | <--- | MI | 0.223 | 0.102 | 2.225 | 0.016 | 0.557 |
| MI2 | <--- | MI | 0.145 | 0.067 | 2.361 | 0.025 | 0.281 |
| MI3 | <--- | MI | 0.327 | 0.132 | 2.257 | 0.032 | 0.438 |
| y1 | <--- | MI1 | 1.000 | —— | —— | —— | 0.732 |
| y2 | <--- | MI1 | 0.865 | 0.096 | 9.076 | *** | 0.728 |
| y3 | <--- | MI1 | 0.932 | 0.105 | 8.943 | *** | 0.721 |
| y4 | <--- | MI1 | 0.953 | 0.092 | 9.134 | *** | 0.708 |
| y5 | <--- | MI1 | 0.883 | 0.104 | 9.047 | *** | 0.719 |
| y6 | <--- | MI2 | 1.000 | —— | —— | —— | 0.752 |
| y7 | <--- | MI2 | 0.932 | 0.103 | 9.072 | *** | 0.727 |
| y8 | <--- | MI2 | 0.953 | 0.107 | 9.085 | *** | 0.713 |
| y9 | <--- | MI2 | 0.892 | 0.105 | 9.082 | *** | 0.692 |
| y10 | <--- | MI3 | 1.000 | —— | —— | —— | 0.783 |
| y11 | <--- | MI3 | 0.798 | 0.094 | 8.221 | *** | 0.676 |
| y12 | <--- | MI3 | 0.965 | 0.104 | 9.298 | *** | 0.772 |
| y13 | <--- | MI3 | 0.976 | 0.116 | 8.361 | *** | 0.703 |
|  |  |  |  |  |  |  |  |

Table 5. Subjective weights of influencing factors of management innovation.

| Indicator | Weight | Indicator | Weight | Indicator | Weight |
| --- | --- | --- | --- | --- | --- |
| MI1 | 0.353 | MI2 | 0.295 | MI3 | 0.341 |
| y1 | 0.263 | y6 | 0.227 | y10 | 0.213 |
| y2 | 0.176 | y7 | 0.222 | y11 | 0.207 |
| y3 | 0.169 | y8 | 0.261 | y12 | 0.269 |
| y4 | 0.217 | y9 | 0.290 | y13 | 0.311 |
| y5 | 0.175 | -- | -- | -- | -- |

Table 6. Objective weights of influencing factors of management innovation.

| Indicator | Weight | Indicator | Weight | Indicator | Weight |
| --- | --- | --- | --- | --- | --- |
| MI1 | 0.434 | MI2 | 0.215 | MI3 | 0.349 |
| y1 | 0.203 | y6 | 0.247 | y10 | 0.261 |
| y2 | 0.206 | y7 | 0.242 | y11 | 0.237 |
| y3 | 0.189 | y8 | 0.231 | y12 | 0.269 |
| y4 | 0.202 | y9 | 0.280 | y13 | 0.234 |
| y5 | 0.200 | -- | -- | -- | -- |
